# Antimicrobial Peptides: a New Frontier in Antifungal Therapy

**DOI:** 10.1128/mBio.02123-20

**Published:** 2020-11-03

**Authors:** Giuseppe Buda De Cesare, Shane A. Cristy, Danielle A. Garsin, Michael C. Lorenz

**Affiliations:** a Department of Microbiology and Molecular Genetics, McGovern Medical School, The University of Texas Health Science Center at Houston, Houston, Texas, USA; b MD Anderson Cancer Center UTHealth Graduate School of Biomedical Sciences, Houston, Texas, USA; Duke University Medical Center

**Keywords:** antifungal drugs, antimicrobial peptides, mycology

## Abstract

Invasive fungal infections in humans are generally associated with high mortality, making the choice of antifungal drug crucial for the outcome of the patient. The limited spectrum of antifungals available and the development of drug resistance represent the main concerns for the current antifungal treatments, requiring alternative strategies. Antimicrobial peptides (AMPs), expressed in several organisms and used as first-line defenses against microbial infections, have emerged as potential candidates for developing new antifungal therapies, characterized by negligible host toxicity and low resistance rates. Most of the current literature focuses on peptides with antibacterial activity, but there are fewer studies of their antifungal properties. This review focuses on AMPs with antifungal effects, including their *in vitro* and *in vivo* activities, with the biological repercussions on the fungal cells, when known. The classification of the peptides is based on their mode of action: although the majority of AMPs exert their activity through the interaction with membranes, other mechanisms have been identified, including cell wall inhibition and nucleic acid binding. In addition, antifungal compounds with unknown modes of action are also described. The elucidation of such mechanisms can be useful to identify novel drug targets and, possibly, to serve as the templates for the synthesis of new antimicrobial compounds with increased activity and reduced host toxicity.

## Abstract (precis)

Invasive fungal infections in humans are generally associated with high mortality, making the choice of antifungal drug crucial for the outcome of the patient. The limited spectrum of antifungals available and the development of drug resistance represent the main concerns for the current antifungal treatments, requiring alternative strategies. Antimicrobial peptides (AMPs), expressed in several organisms and used as first-line defenses against microbial infections, have emerged as potential candidates for developing new antifungal therapies, characterized by negligible host toxicity and low resistance rates.

## INTRODUCTION

The threat of fungal infections is increasing, caused in part by the recent advances in health care therapies that have expanded the population of immunosuppressed patients (1). Unfortunately, the repertoire of effective antifungal agents remains very limited, with only three classes of drugs available for systemic therapy: the polyenes (e.g., amphotericin B), triazoles (e.g., fluconazole), and echinocandins (e.g., caspofungin). A few other drugs (e.g., 5-flucytosine) are available for adjunctive treatments. Furthermore, the limited spectrum and widespread use of antifungal agents have augmented the emergence of drug-resistant strains of *Candida*, *Cryptococcus*, and *Aspergillus* (2–4). In addition, a number of fungal pathogens, including the Mucorales, Candida auris, and some molds, are intrinsically resistant to these agents and difficult to treat at present, emphasizing the need for alternative antifungal strategies.

Antimicrobial peptides (AMPs) were first described in 1939 by Dubos (5), who isolated gramicidin from Bacillus brevis and assessed its antibacterial properties against infections in mice (6). A few years later, in 1948, another peptide family was isolated from Bacillus subtilis, bacillomycin, with low antibacterial effects but remarkable antifungal activity (7). The immunomodulatory functions of AMPs were later described and, together with activity against a broad range of microorganisms, aroused interest in their potential therapeutic applications (8).

While the focus of most of the current studies is on antibacterial peptides, there are many with antifungal properties, and this review will highlight antifungal peptides as important potential additions to the antifungal repertoire. Here, we use the term peptide in a broad sense, including proteins of any length as well as some compounds in which peptides are conjugated to non-amino acid moieties. Furthermore, we focus on the activities and mechanisms of these AMPs; the challenges and benefits of clinical development of these compounds were recently discussed elsewhere (9).

## BIOSYNTHESIS AND STRUCTURE OF AMPs

**FIG 1:**
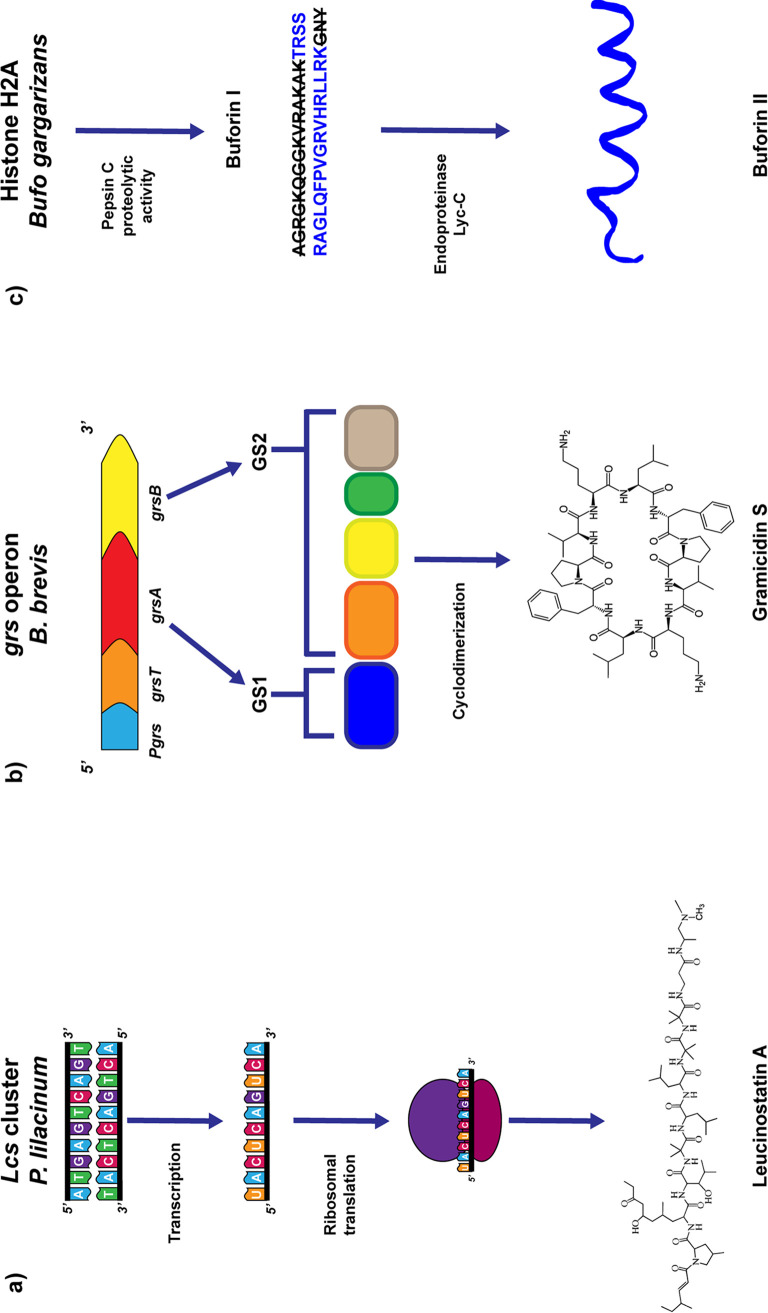
Biosynthesis of antimicrobial peptides. The figure describes the three routes adopted for the production of the AMPs: classical ribosomal synthesis (a), the nonribosomal pathway (b), and the cryptic peptides (c). In ribosomal synthesis, the gene for the AMP is harbored by a cluster that is translated into the mature peptide via ribosomal synthesis of common amino acids, which can undergo structural modifications, such as glycosylation in the case of leucinostatin A. The compounds produced via the nonribosomal route, unlike the previous-described pathway, are assembled by large enzymes, referred to as nonribosomal peptide synthases (NRPS). They incorporate nonproteinogenic amino acids and also catalyze other structural modifications, such as lipidation and cyclization. For example, as shown here, the gramicidin synthases I and II (encoded by *grsA* and *grsB*, respectively), produce the cyclic decapeptide gramicidin S. GS1 modules (blue) consist of three domains in total, responsible for the reactions of adenylation, thiolation, and epimerization. GS2 contains four modules, each containing condensation, adenylation, and thiolation, with a thioesterase at the end. The cryptic peptides originate from the proteolytic digestion of proteins with other functions, such as the histone H2A of the Asian toad. In the toad’s stomach, the enzymatic activity of pepsin C produces buforin I, which in turn is processed by an endopeptidase to generate buforin II.

Antimicrobial peptides are synthesized by three routes (Fig. 1a). Ribosomally coded AMPs, such as human β-defensins and histatins, are typically short (<50 amino acids), cationic (net charge of +2 to +9) amphiphilic peptides found in bacteria, insects, vertebrates, and plants that are produced to fight microbial infections (10). The positive net charge, mainly due to lysine and arginine residues, promotes disruption of phospholipid-rich membranes (11). Other AMPs are generated by nonribosomal peptide synthases (NRPSs) (Fig. 1b) (12, 13), which are mainly found in bacteria (*Actinomycetes* and *Bacilli*, in particular) and filamentous fungi (13). The NRPS-generated AMPs are diverse due to the incorporation of nonproteinogenic amino acids into the sequence (often the d-enantiomers of natural residues) and are often heavily modified through hydroxylation, glycosylation, lipidation, and cyclization (14). Finally, some AMPs are generated through proteolytic cleavage of larger proteins with entirely separate functions and hence are called cryptic peptides (Fig. 1c) (15).

The amphiphilic nature of many of the AMPs determines their structural flexibility. Contact with membranes can induce the formation of secondary structures, such as α-helices, β-sheets, or a mixture of both, that are critical to antimicrobial activity (16). Cyclic peptides can be stabilized through intramolecular disulfide bonds and form helical type II structures, specifically promoted by arginine, histidine, and proline residues (17), while other peptides maintain a linear configuration (18). Some AMPs, such as gramicidin A (19) and tritrpticin (20), are rich in tryptophan, a residue common in transmembrane segments especially close the membrane-water interface. As a result, they induce the formation of ion channels in the target membranes (21). Other peptides, such as the defensins, have a core containing two antiparallel β-sheets with an interposed short turn (22). Another important characteristic of many AMPs is their hydrophobicity, which is responsible for their membranolytic properties and correlates with low toxicity and selectivity toward mammalian cells (23).

## RESISTANCE TO AMPs

AMPs represent one of the possible options to overcome the issue of antimicrobial resistance, partly because they are less susceptible than conventional antibiotics to the evolution of resistance from microorganisms. Although some episodes of resistance against AMPs were described (24–26), the “mutant selection window” (MSW), the concentration range in which selective amplification of single-step, drug-resistant mutants can occur, appears to be narrower than for conventional antibiotics (27). This results in a higher killing rate (28, 29) and lower probability of developing resistance (30). In many cases, the mechanism of action is based on fundamental cellular properties (e.g., negatively charged membranes) that are inherently difficult to change.

## CLASSIFICATION OF ANTIFUNGAL PEPTIDES

**FIG 2:**
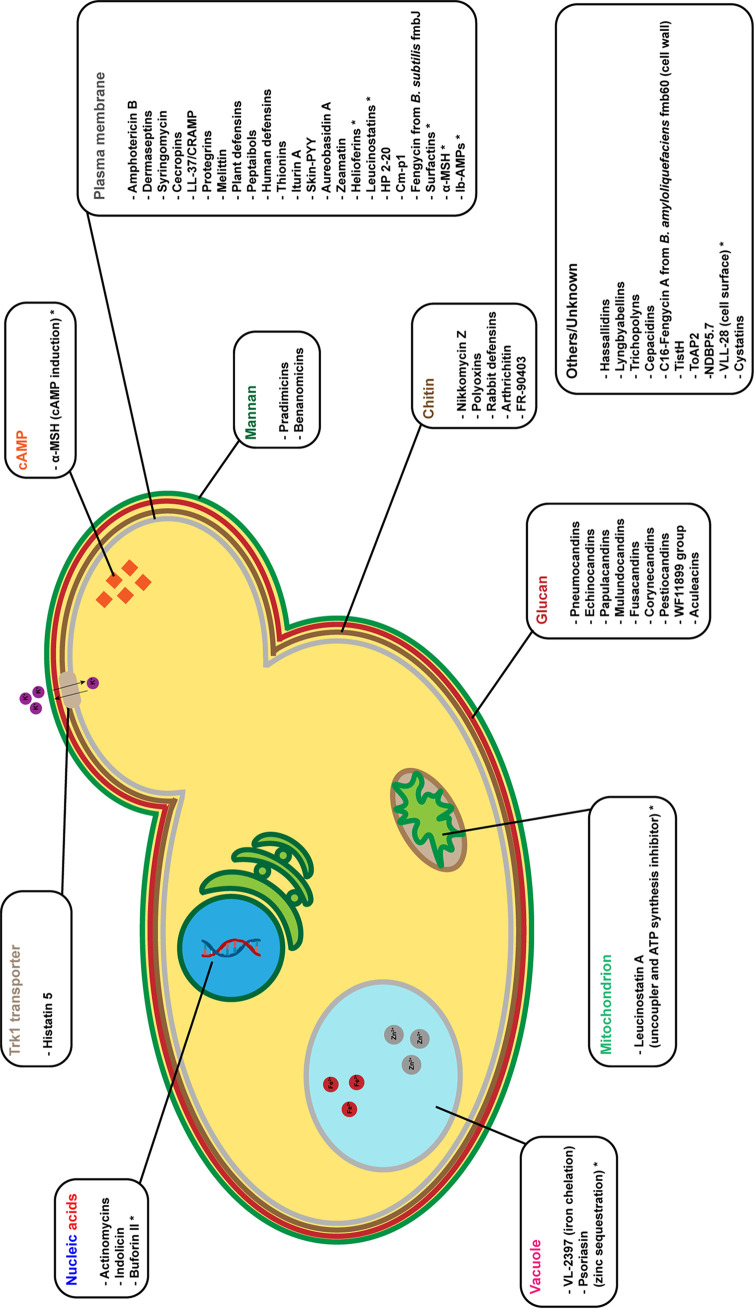
Schematic representation of the targets of the antimicrobial peptides with antifungal activity. The peptides are listed according to the putative target within the fungal cell. The asterisk following some of the peptides indicates the target has only been hypothesized according to the data present in literature.

The following paragraphs describe known antifungal peptides based on the mechanism of action: (i) peptides interacting with membranes, which usually form pores and can have broad-spectrum activity against bacteria as well as well as fungi, (ii) peptides targeting the cell wall, which are usually more specific toward fungi, (iii) nucleic acid inhibitors, and (iv) other peptides, which have either unique or unknown mechanisms of action (31). Relevant features of many of the peptides described below are summarized in Table S1 in the supplemental material and illustrated graphically in Fig. 2.

10.1128/mBio.02123-20.1 TABLE S1 Overview of antifungal peptides. Many of the peptides discussed in the text are summarized in this table, including the putative mechanism of action, source, synthetic route, a link to the chemical structure, and the MIC against C. albicans, A. fumigatus, and C. neoformans. Download Table S1, PDF file, 0.2 MB. Copyright © 2020 Buda De Cesare et al. 2020 Buda De Cesare et al. This content is distributed under the terms of the Creative Commons Attribution 4.0 International license.

## PORE-FORMING PEPTIDES

This class of peptides is the most common among all the AMPs found in nature, characterized by a broad range of activity toward different microorganisms and relatively high toxicity compared to that of other antimicrobials with bacterium- or fungus-specific targets (32). Their mechanisms of action can be described using different models of their effects on the target membranes with which they interact (22).

### Barrel-stave model.

In the “barrel-stave model,” peptides aggregate and form barrel-shaped pores in the membranes, with the peptide helices acting as staves (33). Amphotericin B (AmB) is a polyene macrolide antibiotic produced by Streptomyces nodosus via a polyketide synthase and is, to date, the only natural product with antifungal effects exerted through this mechanism (34). It was long believed that binding of ergosterol in the fungal membrane resulted in pore formation, a rapid leakage of potassium and magnesium, and, ultimately, cell death (35). More recently, a secondary mechanism in which amphotericin physically extracts ergosterol from lipid bilayers was shown to contribute to the fungicidal activity (36, 37). It remains to be seen which of these two mechanisms is more responsible for cidal activity, and it might differ from species to species. Accumulation of reactive oxygen species (ROS) also contributes to the antifungal effect of the drug (38).

### Carpet model.

In the “carpet model,” peptides accumulate on the membrane in a carpet-like manner, attracted by electrostatic interactions (39). At high concentrations of the peptide, the membranes are disrupted and form micelles, with similar effects to treatment with detergents (40). The amphipathic dermaseptin peptides, produced by phyllomedusine frog skin, use this mechanism (39) and are active against fungi, bacteria, protozoa, and viruses (41, 42). In Candida albicans, dermaseptin-S1 inhibits growth and filamentation, confirmed by downregulation of several hypha-associated genes (43).

Also belonging to this group are the lipopeptides of the syringomycin family, secreted by the plant-associated bacterium Pseudomonas syringae, which are particularly active against several filamentous fungi and yeasts, including *Candida*, *Cryptococcus*, and *Aspergillus* strains (44). In addition to the formation of pores, they also induce passive ion fluxes, which generate an electrochemical gradient that alters the pH gradient across the membrane (45).

Cecropins, found in insects, are active against many fungi, including *Aspergillus* and *Fusarium* species (especially *F. moniliforme* [*verticillioides*] and F. oxysporum) (46). In particular, cecropin A induces apoptosis associated with disrupted ion balances and intracellular glutathione redox states in C. albicans (47).

### Toroidal pore model.

In the “toroidal pore model” the peptides insert into the membranes, forming pores and tilting the lipid layers in the fashion of a toroidal hole (48). One of the most studied AMPs in humans, LL-37 (CRAMP in mice), is part of this group (49). This cathelicidin-related peptide is produced by neutrophils and other cells of the innate immune system on epithelial surfaces, where they represent one of the first lines of defense against fungi (50, 51). LL-37 interacts with the cell wall carbohydrates (the main mediators of *Candida* adhesion) and permeabilizes the plasma membrane, with subsequent ROS accumulation (52). Induced expression of CRAMP resulted in a reduction of C. albicans gastrointestinal (GI) colonization and a 50% decrease in mortality in antibiotic-treated mice, demonstrating its key role in innate immunity (53).

Protegrins are cathelicidin-related cationic peptides that form toroidal pores on the plasma membranes of several microorganisms, causing K^+^ imbalance and cell death (54, 55). One of these compounds, the porcine protegrin-1 (PG-1), was particularly active against a broad range of fungi, including several *Candida* species (including drug-resistant strains) and Cryptococcus neoformans, whereas *Aspergillus* species were more resistant (56). In the same study, other cathelicidin peptides were tested, including an ovine (SMAP-29) and two bovine α-helical (BMAP-27 and BMAP-28) peptides, which were as effective as PG-1 but at generally higher MICs, particularly for Candida tropicalis, Candida glabrata, and Candida parapsilosis (56).

Melittin is a cationic amphipathic peptide found in the venom of the honey bee Apis mellifera (57). Its effect on *Candida* involves (toroidal) pore formation (58), caspase- and mitochondrion-dependent apoptotic mechanisms with ROS generation, disruption of mitochondrial membrane potential, and Ca^2+^ release from the endoplasmic reticulum (59, 60). The effect on other fungi is less clear, with other components in the venom potentially responsible for the antimycotic properties (61).

Peptaibols are linear lipopeptides mainly produced as fungal secondary metabolites by NRPSs found in the *Trichoderma*, *Hypocrea*, *Emericellopsis*, and *Boletus* genera, some containing nonproteinogenic amino acids (62). They include many compounds with antifungal activity (reviewed in reference 63). Their mechanism of action is mainly through alteration of membrane permeability by pore formation, which is the reason why they have such a wide range of targets (viruses, protozoa, helminths, and insects) and different degrees of toxicity to mammalian cells dependent on the particular compound (64). Some examples include heptaibin, which has inhibitory activity on the growth of C. albicans, C. neoformans, and Aspergillus fumigatus (65), hyporientalin A, with promising candidacidal activity and relatively low toxicity (66), atroviridins (A, B, C), effective against Aspergillus niger and F. oxysporum (67), longibrachins, displaying anti-*Aspergillus* effects (68), and septocylindrins (A and B), inhibiting C. albicans (69).

## OTHER MEMBRANE-ACTIVE PEPTIDES

A number of antifungal AMPs exert their activity through interactions with membranes, though it is not clear whether they form pores (or what kind of pores) or disrupt membrane integrity through other mechanisms.

Plant defensins are highly stable cysteine-rich peptides with widespread activity against bacteria and fungi that have been extensively studied (see, for instance, reference 61 for a review). Several have antifungal activity, hypothesized to act through either a carpet or toroidal pore model (70). Two members of this family, RsAFP2 and heliomycin, were shown to interact with the glucosylceramides on the plasma membrane of C. albicans and Pichia pastoris, inducing cell death by membrane permeabilization (71). Similarly, NaD1 displays candidacidal activity, activating the high-osmolarity glycerol (HOG) pathway due to ROS production and oxidative damage (72). PvD1, isolated from Phaseolus vulgaris seeds (73), has antifungal activity against *Fusarium* species (F. oxysporum, F. solani, and F. lateritium) and *Candida* species (C. albicans, C. tropicalis, C. parapsilosis, and C. guilliermondii) (74). Other plant defensins with antifungal effects exerted through membrane permeabilization include Dm-AMP1 (75) and the hevein-like Pn-AMP1 and Pn-AMP2 (76).

Pr-1, from pumpkin, inhibits the growth of many fungi, including F. oxysporum, F. solani, and C. albicans, through membrane permeabilization yet did not show hemolytic activity on human red blood cells (77).

Thionins, with a structure similar to that of plant defensins, exert antifungal activity via an unclear mechanism (78). For example, the Capsicum annuum thionin (CaThi) caused membrane permeabilization in C. albicans, C. parapsilosis, and C. tropicalis, where it also induced oxidative stress (79). Yet, the intracellular localization of this peptide in C. albicans and C. tropicalis suggested a possible nuclear target (79). Similar findings were observed with F. solani, where the peptide also showed a synergistic effect with fluconazole (80).

The mechanisms of interaction of the human defensins with membranes are not as well characterized as those of their plant relatives. Some members of this family, which are produced by neutrophils and epithelial cells, have antifungal activity, including β-defensins HBD-1 and HBD-3 and α-defensins HNP-1 and HNP-2 (81). These compounds, detected in salivary glands and secretions, cause membrane permeabilization in fungal pathogens but with different mechanisms: HBD-1 and HBD-2 deploy an ATP-independent mechanism, involving membrane permeabilization, whereas HNP-1 and HNP-2 stimulate cytotoxicity by an efflux of cellular ATP, similarly to histatin 5 (82, 83). Another member of the defensin family, rhesus θ-defensin 1 (RTD-1), displayed fungicidal activity against *Candida* species, including multidrug-resistant C. auris, through cell permeabilization associated with ATP release and intracellular ROS accumulation, similarly to histatins, though it was more rapid and did not require mitochondrial ATP production (84).

Iturin A is a lipopeptide produced by NRPSs in *Bacillus* species and is effective against *Candida*, *Trichosporon*, *Fusarium*, and *Aspergillus* spp. (7, 85, 86) via a pore-dependent mechanism, causing cell wall damage, ROS accumulation, and Hog1-mitogen-activated protein kinase (MAPK) activation (87, 88). Despite potent hemotoxic effects (89, 90), mice infected with C. albicans and treated with iturin A and AmB survived better than those treated with either agent alone (85).

Skin-PYY is an antibacterial and antifungal peptide found on the skin extract of the arboreal frog Phyllomedusa bicolor, displaying similar pharmacological and structural properties as neuropeptide Y (NPY) and polypeptide Y (PPY) found in the brains and intestines, respectively, of multiple vertebrates (91). This amphibian peptide showed moderate effects on *Aspergillus* species (A. fumigatus and A. niger) but higher efficacy against C. albicans, Microsporum canis, Trichophyton rubrum, and Arthroderma simii and appeared to be cidal rather than static (92). Promisingly, a low toxicity was observed for mammalian erythrocytes and macrophages at concentrations severalfold above the MIC for C. albicans (92).

Aureobasidin A (AbA) is a cyclic nonribosomal depsipeptide produced by the black mold Aureobasidium pullulans that exhibits a strong fungicidal activity against *Candida* species, C. neoformans, Blastomyces dermatitidis, and Histoplasma capsulatum but not *Aspergillus* spp. (93–95). The antifungal effect is exerted through the noncompetitive inhibition of the inositol phosphorylceramide (IPC) synthase, responsible for the sphingolipid biosynthesis in fungi and essential for cell viability (94, 96). Toxicity is low due to the absence of the target enzyme in mammalian cells (97). Some fungi, such as A. fumigatus and Aspergillus flavus, are resistant as a result of increased efflux, confirmed by the reduced sensitivity of AbA on the Saccharomyces cerevisiae strain overexpressing Pdr16, a phosphatidylinositol transfer protein (98).

Zeamatin is a 22-kDa peptide isolated from Zea mays seeds, but compounds of the same family have been isolated from Avena sativa, Sorghum bicolor, and Triticum aestivum (99). Its membrane-permeabilizing activity was effective on C. albicans, but *Mucorales* species were resistant (99). Synergistic activity with nikkomycin Z and clotrimazole was detected in a *Candida* vaginitis mouse model (100). It binds β-1,3-glucan, which could be an important step in exerting its membrane-related function and could explain the resistance of *Mucorales* spp., which lack this carbohydrate (101).

HP 2-20 is a cryptic peptide with antifungal and antibacterial properties derived from the N terminus of the ribosomal protein L1 (Rpl1) of Helicobacter pylori (102) and disrupts membranes via pore formation (103). Promisingly, the peptide’s hemolytic activity against mammalian cells was low (103). These effects were tested only on C. albicans and Trichosporon beigelii, with a strong reduction of mortality observed in mice injected with a lethal dose of C. albicans (104, 105).

Cm-p1 is a small hydrophilic peptide identified from the crude extract of the marine snail Cenchritis muricatus (106). The protein from which this is produced is not known, since another sequence (Cm-p2), sharing 70% similarity with Cm-p1, was also found as part of a larger protein in the same organism (106). The low hydrophobicity likely correlates with the lack of toxicity toward human red blood cells and RAW 264.7 cells as well as with the absence of antibacterial activity, although it exhibited broad-spectrum antifungal activity against C. albicans, T. rubrum, A. niger, and F. oxysporum (106). Cm-p5 is a synthetic peptide derived from Cm-p1, with an increased fungistatic effect on C. albicans and C. parapsilosis but with little toxicity to mammalian cell lines (107). The improved activity was due to the affinity toward phospholipids of fungal membranes (phosphatidylserine and phosphatidylethanolamine) but low interaction with ergosterol and mammalian membranes (108).

## AMPs TARGETING THE CELL WALL

### Glucan synthesis inhibitors.

β-Glucan, the major polysaccharide of the fungal cell wall, is a polymer of glucose moieties linked by β-(1,3)- or β-(1,6)-glycosidic bonds that form a branched network conferring strength to the cell wall (109). β-Glucan is of extreme importance for recognition of fungal pathogens by the host innate immune system via dectin-1, a specific receptor for β-(1,3)-glucan, which is essential for fungal recognition and induction of the immune response (110, 111). The echinocandin drugs, in clinical use for almost 20 years, are synthetically optimized derivatives of several nonribosomal AMPs, including pneumocandins and echinocandin B, produced by some fungal species as secondary metabolites (112). They are noncompetitive inhibitors of β-(1,3)-glucan synthase, critical to generating the cell wall in most fungal pathogens (113). The noncompetitive inhibition of the catalytic subunit of this enzyme, encoded by the *GSC* and *FKS* genes, can be overcome by point mutations, found commonly among echinocandin-resistant isolates (114).

Pneumocandins are produced by Zalerion arboricola (115); pneumocandin A0 had potent fungicidal activity against C. albicans but also high hemolytic activity and lacks efficacy against A. flavus, A. fumigatus, C. neoformans, and other *Candida* species (116). Echinocandin B is a fungal lipopeptide isolated from Aspergillus nidulans with potent anti-*Candida* activity (117). To reduce the high toxicity of these compounds on mammalian cells, mainly caused by the hemolytic activity, semisynthetic analogues with much reduced toxicity to mammalian cells but similar antifungal activity, such as cilofungin, have been generated (118).

Three synthetic derivatives emerged from clinical development in the 1990s: caspofungin, anidulafungin, and micafungin (119). These drugs addressed most of the drawbacks of their natural progenitors, providing broader activity and lower toxicity (120–123). The extended-spectrum echinocandins showed fungicidal activity against *Candida* species, including those that are resistant to amphotericin B or fluconazole, and had fungistatic activity against *Aspergillus* species (124). Currently approved echinocandins have limitations related to emerging drug resistance and the need for intravenous delivery. Potential next-generation echinocandins such as SCY-078 (ibrexafungerp; Scynexis, Inc.), an intravenous and orally bioavailable glucan synthase inhibitor, may solve these problems (125). Additionally, it retains *in vitro* activity against echinocandin-resistant isolates of *Candida* species (126, 127).

Other compounds of the same family of echinocandins, which could drive the development of new synthetic antifungals, include papulacandins (128, 129), mulundocandins (130), fusacandins (131), corynecandins (132), pestiocandins (133), and WF11899 (134). Although effective as antifungals (122, 135–137), these lipopeptides were never clinically approved because of lower activity and/or higher toxicity then extended-spectrum echinocandins (138). Aculeacins also belong to this group of antifungal peptides but have lower toxicity as well as lower efficacy against filamentous fungi (139).

### Chitin inhibitors.

Chitin is another essential component of the fungal cell wall. It is composed of *N*-acetylglucosamine moieties connected by β-(1,4) linkages (109), and it is important for cell viability and modulation of the host immune response (140). The amount of chitin in the wall varies according to the cell morphology: for example, C. albicans hyphae can have up to 10 times more *N*-acetylglucosamine than yeast cells (141, 142). Increased content of chitin in the wall has also been linked with resistance to echinocandin drugs (143).

Nikkomycin Z, a dipeptide with a nucleoside sidechain synthesized by Streptomyces tendae, is a competitive inhibitor of chitin synthases (144). The inhibitory activity of nikkomycin Z was demonstrated on a variety of different organisms, including fungal plant pathogens (145). It is active against B. dermatitidis and Coccidioides immitis *in vitro* and in animal models, although a lower efficacy was seen for Histoplasma capsulatum (146–148). It has modest efficacy against A. fumigatus alone but synergizes with echinocandins, generating more successful outcomes (149, 150). Similar results were obtained for the melanized fungus Alternaria infectoria, involved in opportunistic human infections and respiratory allergies (151). Nikkomycin Z also synergizes with caspofungin or micafungin against *Candida* biofilms, in particular, C. albicans and C. parapsilosis (152–154). In fact, the response to caspofungin involves a compensatory increase in chitin synthesis (155–157), and so the ability of nikkomycin Z to target chitin synthesis is a plausible mechanism to explain the synergy between these two drugs (155). Other combinatorial effects were observed with the azoles, in particular, fluconazole and itraconazole against C. albicans and C. parapsilosis and itraconazole versus *C. immitis* and A. fumigatus (147, 158). The efficacy of this AMP in combinatorial therapy with existing antifungal drugs may improve outcomes and reduce the development of resistance. This peptide is under development as an orphan product for treatment of coccidioidomycosis, with phase I studies successfully completed and demonstrating excellent safety in healthy humans (159).

Similar to Nikkomycin Z, the polyoxins (A to L) are nucleoside-tripeptide antibiotics produced by the actinomycete Streptomyces cacaoi that inhibit chitin synthases (160, 161). They are effective not only against phytopathogenic fungi, such as Botrytis cinerea and Alternaria kikuchiana (162), but also against human pathogens such as C. albicans and C. neoformans (163). In particular, polyoxin D causes altered cell morphology in C. albicans, with hyphal inhibition, swollen cells, sensitivity to osmotic changes, and a weakened cell wall, especially at the septum, resulting in an inability to bud (163). Similar effects were observed for C. neoformans, with a greater fungistatic activity detected when incubated with 2 mM polyoxin D, which completely depleted growth (163).

Rabbit defensins NP-3b, NP-4, and NP-1 were shown to be highly active against C. albicans, with NP-5 able to potentiate their effects, whereas only NP-1 was found effective against other medically important fungi, such as C. neoformans, with a much lower MIC for acapsular strains (164). NP-1 also has activity against Rhizopus oryzae as well as hyphae and germinating conidia of *C. immitis* and A. fumigatus, though not resting conidia (165–167). NP-2 also killed A. fumigatus hyphae (165). The action of this peptide family was hypothesized to be related to chitin sequestration, since their preincubation with purified chitin reduced activity against A. fumigatus (165).

Arthrichitin and FR-90403 are produced by Arthrinium phaeospermum and *Kernia* spp., respectively, and, similarly to nikkomycin Z, bind and inhibit chitin synthases Chs1 and Chs2 in C. albicans (168, 169).

### Mannan-binding peptides.

Mannan represents the outermost layer of the fungal cell wall and it is composed of mannan fibrils formed from heavily glycosylated proteins, with α- and β-linked oligomannosyl residues (170). These mannoproteins are involved in many processes, including biofilm formation, virulence, and adhesion (171–173).

One family of secondary metabolites that includes pradimicins and benanomicins targets cell wall mannan. Pradimicins (A to E) are polyketides produced by the actinomycete Actinomadura hibisca (174, 175), whereas benanomicins were isolated from Actinoallomurus spadix (176). They demonstrated a moderate *in vitro* antifungal activity against a broad spectrum of organisms, including *Candida* and *Aspergillus* species and C. neoformans, but a remarkable *in vivo* efficacy in healthy and immunocompromised mice infected with C. albicans, C. neoformans, and A. fumigatus (175, 177). Moreover, benanomicin A was also successful for *in vivo* treatment of Pneumocystis carinii pneumonia (178). Pradimicin A also showed fungicidal effects against pulmonary candidiasis and aspergillosis, vaginal candidiasis, and skin Trichophyton mentagrophytes infection in mice with intravenous or topical treatment (177). The antifungal activity of this family of nonribosomal peptides recognizes d-mannose in a manner similar to that for lectins in the presence on calcium (179, 180), ultimately leading to cell death (181). In S. cerevisiae pradimicin A induced an apoptosis-like cell death through ROS accumulation (182). To date, no ribosomally produced AMPs are known to target mannans.

## NUCLEIC ACID INHIBITORS

The AMPs in this section specifically target nucleic acid biosynthesis and metabolism. Although some of them have been proven to bind DNA, the antimicrobial mechanisms are not completely clear. For example, the activity of buforin II is associated with its specific interaction with the major groove of DNA, but how this is antifungal remains unclear (183). For their capacity to bind nucleic acids, these peptides are also used as antineoplastics (e.g., actinomycin D) and can therefore have significant host effects (e.g., indolicidin). In some cases, the toxicity issue can be overcome by using different formulations, such as liposomes or nanoparticles, which reduces the adverse effects for the host but preserves the activity of the compound.

Various species of *Streptomyces* synthesize actinomycins, a family of chromopeptide lactones with antifungal activity (184). In particular, activity against C. albicans was described for actinomycin D, RSP 01, and RSP 02 (185, 186). Actinomycin D is clinically useful as an antineoplastic and exerts its antifungal function by intercalating the DNA. The other two have been tested with promising results but are not used clinically. Both have structural similarity to actinomycin D and therefore could function in a similar manner (185).

Indolicidin is a tridecapeptide amide of the cathelicidin family, isolated from cytoplasmic granules of bovine neutrophils, with strong antifungal activity against *T. beigelii*, C. albicans, Candida krusei, and A. flavus but has only modest effects on P. carinii and C. glabrata (187–189). Its structure, characterized by 39% tryptophan and 23% proline, was initially thought to target only the cell membrane (189), but later studies showed that it binds DNA and possibly affects DNA processing enzymes and repair mechanisms (190, 191). Although its nonselective activity causes toxicity in humans, liposomal formulations of indolicidin reduced toxicity in mice 100-fold and allowed sufficiently high dosing to successfully treat mice infected systemically with A. fumigatus (192). Other formulations, such as indolicidin-conjugated gold nanoparticles, were effective against fluconazole-resistant C. albicans (193). A graphene-indolicidin nanocomposite formulation treated disseminated candidiasis as effectively as fluconazole in mice (194).

Buforins are cryptic peptides isolated from the stomachs of toads and originate from pepsin-directed proteolysis of histone H2A (195). Buforin II is derived from buforin I and has greater antimicrobial potential, with activity against C. albicans and C. neoformans (196). Initially believed to cause membrane permeabilization (195), further studies demonstrated that buforin II penetrates membranes without forming pores (197), and a possible interaction with nucleic acids was suggested (183).

## OTHER ANTIFUNGAL PEPTIDES

This final section lists the peptides with alternative and incompletely characterized antifungal mechanisms from the sections listed before. Some of them include disruption of the cell integrity (e.g., histatins and cystatins), modulatory properties (e.g., EntV and alpha melanocyte-stimulating hormone [α-MSH]), surface interactions (e.g., surfactins, VLL-28, and psoriasin).

The mechanisms of histatins, and in particular, histatin 5 (Hst5), have been the subject of debate but seem to have an intracellular target (198). Hst5 is a human salivary cationic peptide with fungicidal activity against *Candida* species other than C. glabrata (including C. albicans, C. krusei, C. tropicalis, C. parapsilosis, and C. guilliermondii) (199). In C. albicans, Hst5 binds to Ssa1/2 proteins (the Hsp70 orthologs) present in the cell wall and, once internalized by translocation, induces the formation of reactive oxygen species (ROS) within the cell and the efflux of ATP and ions in a manner dependent on the plasma membrane Trk1 potassium transporter (200–203). Hst5-induced osmotic stress also contributes to cytotoxicity. Zinc binding potentiates the cytotoxic effects of Hst5 P113, a 12-amino-acid proteolytic product of Hst5 that retains substantial anti-*Candida* activity (204, 205).

Cystatins are a family of peptides with antifungal properties on *Candida* and *Aspergillus* species (206, 207). These compounds are naturally found in vertebrates, invertebrates, and plants and exert a competitive inhibition on cysteine proteases (208). The inhibitory effect on fungal species is not characterized but seems to be independent from the protease inhibitory activity observed against bacteria (209). The cystatin purified from chicken egg white displayed fungicidal effects on C. albicans, C. parapsilosis, and C. tropicalis, with only milder influence on C. glabrata, in a similar fashion to that of histatin 5 (206). A recombinant amaranth cystatin showed inhibition of spore germination and growth of A. niger and Aspergillus parasiticus (207). The altered cell morphology and organelle integrity suggest a possible correlation of the fungicidal activity with disruption of cell integrity (207).

The cyanobacterial genera *Lyngbya*, *Nostoc*, and *Hassallia* produce hassallidins and lyngbyabellins (e.g., hectochlorin), which are two distinct families of cyclic peptides that showed potent activity against C. albicans and C. krusei (210, 211). Both of these peptide families have significant toxicity in mammalian cells: hectochlorin hyperpolymerizes actin (212), while hassallidin A disrupts membranes (213), and so their potential as therapeutics is limited.

Cepacidines (A_1_ and A_2_), are glycopeptides produced by Burkholderia cepacia displaying antifungal properties superior to those of AmB (31). These glycopeptides were found to be active against several *Candida* species and other fungal pathogens, including C. neoformans, A. niger, *M. canis*, F. oxysporum, and T. rubrum, but the presence of human serum (50%) strongly reduced the antifungal effect, precluding their utilization as antifungals (214).

EntV is a 68-amino-acid AMP produced by Enterococcus faecalis that showed inhibitory effects on biofilm formation for C. albicans, C. tropicalis, C. parapsilosis, and C. glabrata (215). It also causes a strong reduction (≥50%) of preformed C. albicans biofilms. It conferred protection against C. albicans in nematode infection and oropharyngeal candidiasis murine models (215). It is ribosomally produced and undergoes several processing events after secretion (216). However, its mechanism of action is still unclear. It does not affect fungal viability at all, only hyphal morphogenesis, and therefore is considered to have an antivirulence effect (214).

Leucinostatin A, produced by *Penicillium* (*Purpureocillium*) *lilacinum*, is a peptide antibiotic that, despite displaying antifungal activity against *Candida* spp. (including C. albicans, C. krusei, C. tropicalis, and C. guilliermondii) (217), is unsuitable for clinical use due to substantial host toxicity (218). More recently, it has received renewed interest due to its antitrypanosomal and antitumoral activities (219, 220). Two other fungal products, helioferins A and B, synthesized by the parasitic fungus Mycogone rosea, as well as trichopolyns A and B, secreted by Trichoderma polysporum, showed inhibitory activity against *Candida*, but their mode of action is unknown (221, 222) and they exhibit significant cytotoxicity in mammalian cells (222, 223).

Fengycins and surfactins are families of nonribosomal cyclic lipopeptides produced by Bacillus amyloliquefaciens, some of which have antifungal action, especially against C. albicans, C. tropicalis, and some *Rhizopus* and *Fusarium* species (224–226). Reduced growth, spore germination, and germ tube formation were some of the observed effects, and efficacy was enhanced when combined with ketoconazole (225, 227). Furthermore, it was shown that some fengycin compounds were able to remove 25% to 100% of C. albicans biofilms grown on polystyrene plates (228). The mechanisms of action of the various surfactins and fengycins are diverse and not completely understood. Studies with different peptides have suggested they disrupt the membrane or cell wall, inhibit DNA synthesis, or lead to mitochondrial disruption (224, 225, 229). The hypothesis that surfactins are membrane-active substances was also supported by the inhibition of membrane fusion during invasion of epithelial cells by enveloped viruses (230).

The alpha melanocyte-stimulating hormone (α-MSH) is a neuroendocrine-immune regulatory peptide with antimicrobial potential found in mammals as well as in organisms that lack adaptive immunity (231). Its precursor is expressed in phagocytes (232) and epithelia (233), but posttranslational proteolytic processing is required to convert it to the active form (231). While *in vitro* antifungal activity against C. albicans was reported, including reduction of cell viability and germ tube formation (231), others observed only very mild effects on growth (234). Synthetic analogues have shown increased antifungal potency combined with an augmented half-life and only moderate hemolytic activity that would be necessary for realistic clinical use (235). The immunomodulatory effects of α-MSH include the regulation of nitric oxide production in macrophages and reduced chemotaxis in neutrophils (236, 237). Its immunomodulatory and antimicrobial properties could be exploited for treatment of disorders in which inflammation and infection coexist (238).

Ib-AMPs are cysteine-rich AMPs, found in Impatiens balsamina seeds, comprising four closely related peptides (Ib-AMP1 to -4) derived from a single precursor protein (239). The structure, which is only 20 amino acids long, is characterized by intramolecular disulfide bridges important for retaining antifungal activity, as shown for Ib-AMP1 and -4 when tested against C. albicans and A. flavus (240, 241). The mechanism of action is still unknown, but a distinct target in the plasma membrane was hypothesized (240).

Psoriasin is an AMP isolated from skin lesions of patients with psoriasis (242), with orthologues found in amphibians (243) and cattle (244). In fact, it is the most prominent antibiotic peptide found on the skin of these individuals, who are rarely affected by bacterial and fungal infections (245, 246). It is effective *in vivo* in a mouse lung model for A. fumigatus infection and in a guinea pig tinea pedis model for T. rubrum skin infection (242). Furthermore, *in vitro* experiments showed activity against other dermatophytes, such as T. mentagrophytes, *M. canis*, and Epidermophyton floccosum (247), which are currently difficult to treat. The target of this AMP is currently unknown, but its activity was inhibited by elevated zinc, suggesting that this compound interferes with zinc homeostasis and its sequestration could be a possible antimicrobial mechanism (242, 248). Surprisingly, this AMP was not effective in killing C. albicans, although it was able to bind β-glucan and inhibit adhesion to surfaces (249).

VL-2397 (formerly ASP2397) is a cyclic hexapeptide isolated from Acremonium persicinum, which exhibited potent *in vitro* fungicidal activity against *Aspergillus* species (var. *fumigatus*, *nidulans*, *flavus*, and *terreus*), C. neoformans, C. glabrata, Candida kefyr, and Trichosporon asahii (250). Its mechanism of action is related to its structure, which resembles ferrichrome, an iron-chelating siderophore, and results in arrest of hyphal elongation. In a model of invasive pulmonary aspergillosis, immunocompromised mice treated with this compound survived longer and had lower lung fungal burdens than control animals (251, 252). It was also efficacious against invasive candidiasis in neutropenic mice caused by drug-resistant C. glabrata (253). Moreover, a phase I study showed promising results regarding its safety and tolerability in healthy individuals (254).

VLL-28 is the first AMP isolated from the archaeal kingdom and is produced by proteolysis of a transcription factor of Sulfolobus islandicus (255). This cryptic peptide displays the same chemophysical and functional properties of typical AMPs, including broad-spectrum antibacterial and antifungal activities, particularly against C. albicans and C. parapsilosis via inhibition of growth and biofilm formation, including reduction of preformed biofilms (256). The antifungal mechanism is unknown, although the peptide seems to interact with the cell surface, either the wall and/or membrane, though in bacteria, it binds nucleic acids in the cytoplasm (255).

Scorpion venom is the source of a great number of peptides with antifungal activity, with similar characteristics, such as cationic character and structural flexibility (257). TistH is an alpha-helical peptide found in the venom of the scorpion Tityus stigmurus, part of the hypotensin family (258). It has moderate effects on C. albicans, C. tropicalis, T. rubrum, and A. flavus, with great strain-to-strain variability in susceptibility. It is characterized by the absence of cytotoxicity and *in vivo* inflammatory activity (258), but its mechanism is otherwise unknown. The maximum efficacy of this compound was obtained by incorporation within chitosan particles, providing improved antifungal effects (including on cell viability and biofilm formation), a prolonged released profile, and maintenance of biocompatibility (259). ToAP2 and NDBP5.7 are another two peptides produced by the scorpions Tityus obscurus and Opisthacanthus cayaporum, respectively, with remarkable antifungal activity on C. albicans (260). Some of the effects include membrane permeabilization with cell wall alteration, disruption of ultracellular structure, and inhibition of filamentation on early phase and mature biofilms (260). The therapeutic potential of the ToAP2 compound was supported by the protective activity in Galleria mellonella infection model and its synergism with AmB and fluconazole (260).

## FUTURE PROSPECTS

The current antimycotic therapies are limited by the restricted choice of available compounds, and the increasing resistance of fungal pathogen further narrows the therapeutic options. The diversity of AMPs expands the development space for future antifungal therapeutics. Although escape strategies from the antifungal activity of the AMPs were described, including secretion of peptide effectors, AMP efflux pumps, and regulation of signaling pathways (261), they are, in fact, less prone to the development of resistance due to the rapid effect and the pharmacodynamic properties in comparison to conventional drugs (262).

However, the challenges to antimicrobial drug development are well known, as recently reviewed (9), and there are only a few examples of antifungal peptides being brought to clinical trials, including nikkomycin Z, aureobasidin A, and VL-2397 (263). The biochemical and cell biological processes of the fungal pathogens are more closely related to those of the host compared to those of bacteria, representing one of the main scientific challenges of antifungal drug development but also presenting an opportunity that these complex molecules might be more specific and thus some may have low host toxicity. In addition to these scientific challenges, the design of clinical trials for antifungals poses several difficulties, including the costs related to the difficulty of finding eligible patients in a timely and unequivocal fashion (264).

As with small molecule antifungals, there is significant potential to use *in silico* peptide optimization to either design novel peptides *de novo* or improve naturally occurring ones (265). Further investigations on AMPs and their mechanisms of action are therefore required to elucidate novel antifungal strategies and pathogenicity mechanisms. The advancements in computational approaches, with predictions of drug target (266) and resistance development (267), and the design of synthetic and semisynthetic peptides (268) represent a valid and inexpensive strategy to reduce the costs related to antifungal compound discovery and design.
