# Supplementary material for: Antimicrobial Peptides: a New Frontier in Antifungal Therapy
Source: mBio. 2020 Nov 3;11(6):e02123-20. doi: 10.1128/mBio.02123-20 (PMC7642678; doi:10.1128/mBio.02123-20)
Supplement: TABLE S1 [file mBio.02123-20-st001.pdf]

*Table 1. Overview of antifungal peptides.* The description includes, in order: the putative mechanism of action; source; synthetic route; chemical structure; minimum inhibitory concentration (MIC) for *C. albicans, A. fumigatus* and *C. neoformans*; references.

| Peptide | Putative mechanism | Source | Synthetic route (MW) | Chemical Structure | MICs (µg/ml) | | | References |
|---|---|---|---|---|---|---|---|---|
| | | | | | ***Candida albicans*** | ***Aspergillus fumigatus*** | ***Cryptococcus neoformans*** | |
| **Pore forming** | | | | | | | | |
| **Amphotericin B** | Pore forming - Barrel and Stave model | *Streptomyces nodosus* | Non ribosomal (0.92 kDa) | Yamamoto et al, 2019 (1) | 0.25 - 2 | 0.5 - 4 | 0.12 - 2 | (2–5) |
| **Dermaseptins - Dermaseptin S1** | Pore forming - Carpet model | *Phyllomedusa sauvagei* - waxy monkey tree frog | Ribosomal (3.45 kDa) | PubChem: 16130489 | 10 - 69. | 30 | 0.5 - 1 | (6, 7) |
| **Syringomycin - syringomycin E** | Pore forming - Carpet model | *Pseudomonas syringae* | Non ribosomal (1.22 kDa) | Sorensen et al, 1996 (8) | 2.5 - 5 | 10 - 20. | 2.5 - 10 | (8) |
| **Cecropins - cecropin A** | Pore forming - Carpet model | *Hyalophora cecropia* - cecropia moth | Ribosomal (4 kDa) | Lee et al, 2013 (9) | 1.3 | 50 | NA | (9, 10) |
| **LL-37 / CRAMP** | Pore forming - toroidal pore model | *Homo sapiens / Mus musculus* | Ribosomal (4.49 kDa) | NMR from Wang 2008 (11) | >250<br>64 | 38 | NA | (12, 13) |
| **Protegrins - Protegrin-1** | Pore forming - toroidal pore model | *Sus scrofa domesticus* - domestic pig | Ribosomal (2.15 kDa) | Fahrnerl et al, 1996 (14) | 4 | 14 - 64 | 1 - 4. | (15) |
| **Melittin** | Pore forming - toroidal pore model | Venom of honeybee *Apis mellifera* | Ribosomal (2.84 kDa) | Ramirez et al, 2019 (16) | 3.5 - 91 | 3.5 | NA | (17–19) |

| | | | | | | | | |
|---|---|---|---|---|---|---|---|---|
| **Iturin A** | Pore dependent, but causes cell wall damage, ROS accumulation, and Hog1-MAPK activation | *Bacillus spp* | Non ribosomal (1.04 kDa) | ChemIDplus: 52229-90-0 | 32 | NA | NA | (20) |
| **Skin-PYY** | Pore formation | *Phyllomedusa bicolor* - giant monkey frog | Ribosomal (4.3 kDa) | RCSB PDB: 2DEZ | 25 | 100 | 25 | (21) |
| **Aureobasidin A** | Pore formation -non-competitive inhibition of IPC | *Aureobasidium pullulans* | Non ribosomal (1.1 kDa) | Takesako et al, 1993 (22) | <0.04 - 4 | >50 | 0.31 - 0.63 | (22–24) |
| **Zeamatin** | Membrane permeabilization | *Zea mays* (corn) seeds | Ribosomal (22 kDa) | RCSB PDB: 1DU5 | NA | NA | NA | |
| **Peptaibols -** heptaibin | Alteration of membrane permeability by pore formation | *Emericellopsis sp BAUA8289* | Non ribosomal (1.56 kDa) | De Zotti et al, 2011 (25) | 32 | 13 | 32 | (26) |
| **Thionins - CaThi** | Membrane permeabilization through unclear mechanism | *Capsicum annuum* - chili pepper plant | Ribosomal (5 kDa) | Predicted structure from Nikte et al, 2020 (27) | 10 | NA | NA | (28) |
| **Plant Defensins** | | | | | | | | |

| | | | | | | | | |
|---|---|---|---|---|---|---|---|---|
| **RsAFP2** | Membrane permeabilization through glucosylceramides interaction | *Raphanus sativus* - Radish | Ribosomal (5.74 kDa) | RCSB PDB: 2N2R | 14.35 | NA | NA | (29) |
| **Human Defensins** | | | | | | | | |
| **RTD-1** | Cell permeabilization associated with ATP release and intracellular ROS accumulation | *Macaca mulatta* - Rhesus macaque | Ribosomal (2.08 kDa) | RCSB PDB: 1HVZ | 6.25 - 12.5 | NA | <5 | (30, 31) |
| **β-1,3-glucan synthase** | | | | | | | | |
| **Pneumocandins - Pneumocandin A0** | Non-competitive inhibition of β-1,3-glucan synthase | *Zalerion arboricola* | Non ribosomal (1.08 kDa) | ChemBook: 539823-80-8 | "Candida spp.": 0.06 - 8.0 | "Aspergillus spp.": >128 | NA | (32) |
| **Echinocandin B** | Noncompetitive inhibition of β-1,3-glucan synthase | *Aspergillus nidulans* | Non ribosomal (1.06 kDa) | Hashizume &Nishimura, 2008, figure 36 (33) | "Candida spp.": 0.01 | "Aspergillus spp.": >128 | NA | (32) |
| **Aculeacin A** | Noncompetitive inhibition of β-1,3-glucan synthase | *Aspergillus aculeatus* | Non ribosomal (1.04 kDa) | PubChem: 14315169 | 0.04 - 0.2 | >80 | >80 | (34, 35) |
| **Other glucans** | | | | | | | | |
| **Nikkomycin Z** | Competitive inhibition of chitin synthases | *Streptomyces tendae* | Non ribosomal (0.49 kDa) | PubChem: 456557 | 4 | >64 | >64 | (36) |

| | | | | | | | | |
|---|---|---|---|---|---|---|---|---|
| **Polyoxins - Polyoxin D** | Inhibition of chitin synthases | *Streptomyces cacaoi* | Non ribosomal (0.52 kDa) | PubChem: 72476 | 0.26 | >16 | 0.26 | (37, 38) |
| **Rabbit Defensins - NP-1** | Chitin sequestration | Rabbit | Ribosomal (3.44 kDa) | PubChem: 16130476 | 2.3 | NA | 3.75 - 15 | (39, 40) |
| **Pradimicins** - Pradimicin A | D-mannose recognition | *Actinomadura hibisca* | Non ribosomal (0.84 kDa) | Oki et al, 1988 (41) | 6.3 | 3.1 | 0.8 | (41) |
| **Benanomicins - Benanomicin A** | D-mannose recognition | *Actinomadura spadix* | Non ribosomal (0.83 kDa) | Kondo et al, 1990 (43) | 6.25 | 3.13 - 6.25 | 1.56 | (44) |
| **Others** | | | | | | | | |
| **Actinomycins - Actinomycin D** | DNA intercalation | *Streptomyces spp* | Non ribosomal (1.25 kDa) | Rathod et al, 2018 (45) | 0.062 | NA | NA | (45) |
| **Indolicidin** | DNA binding | Cytoplasmic granules of bovine neutrophils | Ribosomal (0.9 kDa) | Rozek et al, 2000 (46) | 25 - 50 | NA | 25 | (47, 48) |
| **Histatins - Histatin 5** | Unidentified intracellular target responsible for ROS formation and ATP efflux | Human saliva | Ribosomal (3.04 kDa) | ChemBook | 4 - 16. | 54.65 | 2.1 | (49) |
| **Hassallidins - hassallidin A** | Membrane disruption | *Hassallia spp* and other cyanobacteria | Non ribosomal (1.4 kDa) | Neuhof et al, 2005 (50) | 4 - 4.8 | 4.8 | 4 | (50, 51) |
| **Lyngbyabellins** - hectochlorin | Hyperpolymerization of actin | *Lyngbya majuscula* (filamentous cyanobacteria) | Non ribosomal (0.67 kDa) | Marquez et al, 2002 (52) | 10 µg/disk | NA | NA | (52) |

| | | | | | | | | |
|---|---|---|---|---|---|---|---|---|
| **Cepacidines - Cepacidine A** | Unknown | *Burkholderia cepacia* | Non ribosomal (1.22 kDa) | Lee et al., 1994 (53) | 0.391 | NA | 0.025 | (53) |
| **EntV** | Unknown | *Enterococcus faecalis* | Ribosomal (68 kDA) | NA | 0.02 (biofilm inhibition); 6.8 (hyphal inhibition) | NA | NA | (54) |
| **Leucinostatin A** | Possible plasma membrane interaction | *Penicillium lilacinum* | Non ribosomal (1.22 kDa) | Ishiyama et al, 2009 (55) | 1.25 - 10 | NA | 1 | (56) |
| **Fengycins - C-16-Fengycin A** | Cell wall interaction | *Bacillus amyloliquefaciens* | Non ribosomal (1.46 kDa) | H. Desmyttere et al 2019 (57) | 32 | NA | NA | (58) |
| **α-MSH** | Possible membrane permeabilization and cAMP induction | Vertebrates | Ribosomal (1.66 kDa) | PubChem: 44273719 | >166.5 | NA | NA | (59) |
| **Ib-AMP1** | Unknown | *Impatiens balsamina* seeds | Ribosomal (3.03 kDa) | PubChem: 16132245 | 15.2 | NA | NA | (60) |
| **Psoriasin** | Interference with zinc homeostasis | Skin lesions of patient with psoriasis | Ribosomal (11.46 kDa) | Brodersen et al, 1998 (61) | >229 | 22.9 | NA | (62, 63) |
| **VL-2397** | Resemblance to ferrichrome | *Acremonium persicinum MF -347833* | Ribosomal (0.91 kDa) | Kovanda et al, 2019 (64) | >16 | 0.06 - 0.5 | 2 | (65) |
| **TistH** | Unknown | Venom of the scorpion *Tityus stigmurus* | Ribosomal (2.62 kDa) | Machado et al, 2015 (66) | 128 | NA | NA | (67) |

| | | | | | | | | |
|---|---|---|---|---|---|---|---|---|
| **ToAP2** | Unknown | Venom of the scorpion *T. obscurus* | Ribosomal (3 kDa) | Marques-Neto et al, 2018 (68) | 0.037 | NA | 0.018 - 0.037 | (69) |
| **Cryptic** | | | | | | | | |
| **VLL-28** | Unknown, possible interaction with cell surface | Stf76 of *Sulfolobus islandicus* | Cryptic (3.54 kDa) | Notomista et al, 2015 (70) | 88.5 | NA | NA | (71) |
| **Buforin 2** | Possible interaction with nucleic acids | H2A histone of toads | Cryptic (2.43 kDa) | Park et al, 2000 (72) | 1 | NA | 1 | (72) |
| **HP2-20** | Membrane disruption via pore formation | Rpl1 of *Helicobacter pylori* | Cryptic (2.32 kDa) | RCSB PDB: 1P0G | 29 - 58 | 58 | >116 | (73–75) |
| **Cm-p1** | Plasma membrane interaction | *Cenchritis muricatus* - marine snail | Cryptic (1.22 kDa) | RCSB PDB: 6CTG | 32 - 64 | 256 | 128 | (76, 77) |

## References

1. Yamamoto T, Umegawa Y, Yamagami M, Suzuki T, Tsuchikawa H, Hanashima S, Matsumori N, Murata M. 2019. The Perpendicular Orientation of Amphotericin B Methyl Ester in Hydrated Lipid Bilayers Supports the Barrel-Stave Model. Biochemistry 58:2282–2291.
2. Reichert-Lima F, Busso-Lopes AF, Lyra L, Peron IH, Taguchi H, Mikami Y, Kamei K, Moretti ML, Schreiber AZ. 2016. Evaluation of antifungal combination against *Cryptococcus* spp. Mycoses 59:585–593.
3. Archibald LK, Tuohy MJ, Wilson DA, Nwanyanwu O, Kazembe PN, Tansuphasawadikul S, Eampokalap B, Chaovavanich A, Reller LB, Jarvis WR, Hall GS, Procop GW. 2004. Antifungal Susceptibilities of *Cryptococcus neoformans*. Emerg Infect Dis 10:143–145.
4. Guinea J, Peláez T, Alcalá L, Ruiz-Serrano MJ, Bouza E. 2005. Antifungal susceptibility of 596 *Aspergillus fumigatus* strains isolated from outdoor air, hospital air, and clinical samples: Analysis by site of isolation. Antimicrob Agents Chemother 49:3495–3497.
5. Meletiadis J, Antachopoulos C, Stergiopoulou T, Pournaras S, Roilides E, Walsh TJ. 2007. Differential fungicidal activities of amphotericin B and voriconazole against *Aspergillus* species determined by microbroth methodology. Antimicrob Agents Chemother 51:3329–3337.
6. Mor A, Nicolas P. 1994. Isolation and structure of novel defensive peptides from frog skin. Eur J Biochem 219:145–154.

7. Belmadani A, Semlali A, Rouabhia M. 2018. Dermaseptin-S1 decreases *Candida* albicans growth, biofilm formation and the expression of hyphal wall protein 1 and aspartic protease genes. J Appl Microbiol 125:72–83.

8. Sorensen KN, Kim KH, Takemoto JY. 1996. In vitro antifungal and fungicidal activities and erythrocyte toxicities of cyclic lipodepsinonapeptides produced by *Pseudomonas syringae pv. syringae*? Antimicrob Agents Chemother 40:2710–2713.

9. Lee E, Jeong KW, Lee J, Shin A, Kim JK, Lee J, Lee DG, Kim Y. 2013. Structure-activity relationships of cecropin-like peptides and their interactions with phospholipid membrane. BMB Rep 46:282–287.

10. Andrä J, Berninghausen O, Leippe M. 2001. Cecropins, antibacterial peptides from insects and mammals, are potently fungicidal against *Candida albicans*. Med Microbiol Immunol 189:169–173.

11. Wang G. 2008. Structures of human host defense cathelicidin LL-37 and its smallest antimicrobial peptide KR-12 in lipid micelles. J Biol Chem 283:32637–32643.

12. Luo Y, McLean DTF, Linden GJ, McAuley DF, McMullan R, Lundy FT. 2017. The Naturally Occurring Host Defense Peptide, LL-37, and Its Truncated Mimetics KE-18 and KR-12 Have Selected Biocidal and Antibiofilm Activities Against *Candida albicans, Staphylococcus aureus*, and *Escherichia coli In vitro*. Front Microbiol 8:544.

13. Shin SY, Kang SW, Lee DG, Eom SH, Song WK, Kim J Il. 2000. CRAMP analogues having potent antibiotic activity against bacterial, fungal, and tumor cells without hemolytic activity. Biochem Biophys Res Commun 275:904–909.

14. Fahrnerl RL, Dieckmannl T, Harwig3 SS, Lehrer RI, Eisenbergl D, Feigot& J. Solution structure of protegrin-1, a broad-

spectrum antimicrobial peptide from porcine leukocytes.

15. Benincasa M, Scocchi M, Pacor S, Tossi A, Nobili D, Basaglia G, Busetti M, Gennaro R. 2006. Fungicidal activity of five cathelicidin peptides against clinically isolated yeasts. J Antimicrob Chemother 58:950–9.

16. Ramirez LS, Pande J, Shekhtman A. 2019. Helical Structure of Recombinant Melittin. J Phys Chem B 123:356–368.

17. Jia F, Wang J, Peng J, Zhao P, Kong Z, Wang K, Yan W, Wang R. 2018. The in vitro, in vivo antifungal activity and the action mode of Jelleine-I against *Candida* species. Amino Acids 50:229–239.

18. Lee J, Hwang JS, Hwang IS, Cho J, Lee E, Kim Y, Lee DG. 2012. Coprisin-induced antifungal effects in *Candida albicans* correlate with apoptotic mechanisms. Free Radic Biol Med 52:2302–2311.

19. Sung WS, Lee DG. 2008. Pleurocidin-derived antifungal peptides with selective membrane-disruption effect. Biochem Biophys Res Commun 369:858–861.

20. Lei S, Zhao H, Pang B, Qu R, Lian Z, Jiang C, Shao D, Huang Q, Jin M, Shi J. 2019. Capability of iturin from *Bacillus subtilis* to inhibit *Candida albicans in vitro* and *in vivo*. Appl Microbiol Biotechnol 103:4377–4392.

21. Vouldoukis I, Shai Y, Nicolas P, Mor A. 1996. Broad spectrum antibiotic activity of skin-PYY. FEBS Lett 380:237–240.

22. Takesako K, Kuroda H, Inque T, Haruna F, Yohikawa Y, Kato I, Uchida K, Hiratani T, Yamaguchi H. 1993. Biological properties of aureobasidin A, a cyclic depsipeptide antifungal antibiotic. J Antibiot (Tokyo) 46:1414–1420.

23. Zhong W, Jeffries MW, Georgopapadakou NH. 2000. Inhibition of inositol phosphorylceramide synthase by aureobasidin A in

*Candida* and *Aspergillus* species. Antimicrob Agents Chemother 44:651–653.

24. Tan HW, Tay ST. 2013. The inhibitory effects of aureobasidin A on *Candida* planktonic and biofilm cells. Mycoses 56:150–156.

25. De Zotti M, Biondi B, Peggion C, Park Y, Hahm K-S, Formaggio F, Toniolo C. 2011. Synthesis, preferred conformation, protease stability, and membrane activity of heptaibin, a medium-length peptaibiotic. J Pept Sci 17:585–594.

26. Ishiyama D, Satou T, Senda H, Fujimaki T, Honda R, Kanazawa S. 2000. Heptaibin, a novel antifungal peptaibol antibiotic from *Emericellopsis sp*. BAUA8289. J Antibiot (Tokyo). Japan Antibiotics Research Association.

27. Nikte S, Gahankari A, Mulla J, Sengupta D, Joshi M, Tamhane V. 2020. In vitro and in silico studies on membrane interactions of diverse *Capsicum annuum* flower γ-thionin peptides. Proteins Struct Funct Bioinforma 88:227–236.

28. Taveira GB, Carvalho AO, Rodrigues R, Trindade FG, Da Cunha M, Gomes VM. 2016. Thionin-like peptide from *Capsicum annuum* fruits: mechanism of action and synergism with fluconazole against *Candida* species. BMC Microbiol 16:12.

29. Thevissen K, Warnecke DC, François IEJA, Leipelt M, Heinz E, Ott C, Zähringer U, Thomma BPHJ, Ferket KKA, Cammue BPA. 2004. Defensins from Insects and Plants Interact with Fungal Glucosylceramides. J Biol Chem 279:3900–3905.

30. Tang YQ, Yuan J, Ösapay G, Ösapay K, Tran D, Miller CJ, Ouellette AJ, Selsted ME. 1999. A cyclic antimicrobial peptide produced in primate leukocytes by the ligation of two truncated α-defensins. Science (80- ) 286:498–502.

31. Basso V, Garcia A, Tran DQ, Schaal JB, Tran P, Ngole D, Aqeel Y, Tongaonkar P, Ouellette AJ, Selsteda ME. 2018.

Fungicidal Potency and Mechanisms of -Defensins against Multidrug-Resistant *Candida* Species. Antimicrob Agents Chemother 62.

32. Datta KK, Patil AH, Patel K, Dey G, Madugundu AK, Renuse S, Kaviyil JE, Sekhar R, Arunima A, Daswani B, Kaur I, Mohanty J, Sinha R, Jaiswal S, Sivapriya S, Sonnathi Y, Chattoo BB, Gowda H, Ravikumar R, Prasad TSK. 2016. Proteogenomics of *Candida tropicalis*-An Opportunistic Pathogen with Importance for Global Health. OMICS 20:239–247.
33. Hashizume H, Nishimura Y. 2008. Cyclic lipopeptide antibiotics, p. 693–751. *In* Studies in Natural Products Chemistry. Elsevier.
34. Iwata K, Yamamoto Y, Yamaguchi H, Hiratani T. 1982. In vitro studies of aculeacin A, a new antifungal antibiotic. J Antibiot (Tokyo) 35:203–209.
35. Hawser S, Islam K. Mimimum fungicidal concentration.
36. Li RK, Rinaldi MG. 1999. In vitro antifungal activity of nikkomycin Z in combination with fluconazole or itraconazole. Antimicrob Agents Chemother 43:1401–1405.
37. Becker JM, Covert NL, Shenbagamurthi P, Steinfeld AS, Naider F. 1983. Polyoxin D inhibits growth of zoopathogenic fungi. Antimicrob Agents Chemother 23:926–929.
38. Verwer PEB, Van Duijn ML, Tavakol M, Bakker-Woudenberg IAJM, Van De Sande WWJ. 2012. Reshuffling of *Aspergillus fumigatus* cell wall components chitin and β-glucan under the influence of caspofungin or nikkomycin Z alone or in

combination. Antimicrob Agents Chemother 56:1595–1598.

39. Alcouloumre MS, Ghannoum MA, Ibrahim AS, Selsted ME, Edwards JE. 1993. Fungicidal properties of defensin NP-1 and activity against *Cryptococcus neoformans in vitro*. Antimicrob Agents Chemother 37:2628–2632.

40. Selsted ME, Szklarek D, Ganz T, Lehrer RI. 1985. Activity of rabbit leukocyte peptides against *Candida albicans*. Infect Immun 49:202–206.

41. Oki T, Konishi M, Tomatsu K, Tomita K, Saitoh KI, Tsunakawa M, Nishio M, Miyaki T, Kawaguchi H. 1988. Pradimicin, A novel class of potent antifungal antibiotics. J Antibiot (Tokyo) 41:1701–1704.

42. Gomi S, Uotani K, Inouye S. 1991. Isolation of new minor benanomicins. J Antibiot (Tokyo) 44:123–129.

43. Kondo S, Gomi S, Uotani K, Inouye S. 1990. Isolation of new minor benanomicins. J Antibiot (Tokyo) 44:123–129.

44. Kumagai H, Umekita M, Sawa R, Takahashi Y, Arisawa A, Isshiki K, Nishimura Y, Akamatsu Y. 2008. Generation of new benanomicin analogues by biotransformation using *Escherichia coli* expressing actinomycete cytochrome P450. J Antibiot (Tokyo) 61:394–399.

45. Rathod BB, Korasapati R, Sripadi P, Reddy Shetty P. 2018. Novel actinomycin group compound from newly isolated *Streptomyces sp.* RAB12: isolation, characterization, and evaluation of antimicrobial potential. Appl Microbiol Biotechnol 102:1241–1250.

46. Rozek A, Friedrich CL, Hancock REW. 2000. Structure of the bovine antimicrobial peptide indolicidin bound to

dodecylphosphocholine and sodium dodecyl sulfate micelles. Biochemistry 39:15765–15774.

47. Rahimi H, Roudbarmohammadi S, Hamid Delavari H, Roudbary M. 2019. Antifungal effects of indolicidin-conjugated gold nanoparticles against fluconazole-resistant strains of *Candida albicans* isolated from patients with burn infection. Int J Nanomedicine 14:5323–5338.

48. Giacometti A, Cirioni O, Barchiesi F, Caselli F, Scalise G. 1999. In-vitro activity of polycationic peptides against *Cryptosporidium parvum, Pneumocystis carinii* and yeast clinical isolates. J Antimicrob Chemother 44:403–406.

49. Helmerhorst EJ, Reijnders IM, Van 'T Hof W, Simoons-Smit I, Veerman ECI, Amerongen AVN. 1999. Amphotericin B- and fluconazole-resistant *Candida spp., Aspergillus fumigatus*, and other newly emerging pathogenic fungi are susceptible to basic antifungal peptides. Antimicrob Agents Chemother 43:702–704.

50. Neuhof T, Schmieder P, Preussel K, Dieckmann R, Pham H, Bartl F, Von Döhren H. 2005. Hassallidin A, a Glycosylated Lipopeptide with Antifungal Activity from the *Cyanobacterium Hassallia sp*. J Nat Prod 68.

51. Neuhof T, Seibold M, Thewes S, Laue M, Han CO, Hube B, von Döhren H. 2006. Comparison of susceptibility and transcription profile of the new antifungal hassallidin A with caspofungin. Biochem Biophys Res Commun 349:740–749.

52. Marquez BL, Watts KS, Yokochi A, Roberts MA, Verdier-Pinard P, Jimenez JI, Hamel E, Scheuer PJ, Gerwick WH. 2002. Structure and absolute stereochemistry of hectochlorin, a potent stimulator of actin assembly. J Nat Prod 65:866–871.

53. Lee C hoon, Kim S, Hyun B, Suh J woo, Yon C, Kim C, Lim Y, Kim C. 1994. Cepacidine A, A novel antifungal antibiotic

produced by *Pseudomonas cepacia*. I. Taxonomy, production, isolation and biological activity. J Antibiot (Tokyo) 47:1402–1405.

54. Graham CE, Cruz MR, Garsin DA, Lorenz MC. 2017. *Enterococcus faecalis* bacteriocin EntV inhibits hyphal morphogenesis, biofilm formation, and virulence of *Candida albicans*. Proc Natl Acad Sci U S A 114:4507–4512.

55. Ishiyama A, Otoguro K, Iwatsuki M, Namatame M, Nishihara A, Nonaka K, Kinoshita Y, Takahashi Y, Masuma R, Shiomi K, Yamada H, Omura S. 2009. *In vitro* and *in vivo* antitrypanosomal activities of three peptide antibiotics: Leucinostatin A and B, alamethicin i and tsushimycin. J Antibiot (Tokyo) 62:303–308.

56. De Lucca AJ, Walsh TJ. 1999. Antifungal peptides: Novel therapeutic compounds against emerging pathogens. Antimicrob Agents Chemother. American Society for Microbiology (ASM).

57. Desmyttere H, Deweer C, Muchembled J, Sahmer K, Jacquin J, Coutte F, Jacques P. 2019. Antifungal Activities of *Bacillus subtilis* Lipopeptides to Two *Venturia inaequalis* Strains Possessing Different Tebuconazole Sensitivity. Front Microbiol 10:2327.

58. Liu Y, Lu J, Sun J, Zhu X, Zhou L, Lu Z, Lu Y. 2019. C16-Fengycin A affect the growth of *Candida albicans* by destroying its cell wall and accumulating reactive oxygen species. Appl Microbiol Biotechnol 103:8963–8975.

59. Grieco P, Carotenuto A, Auriemma L, Limatola A, Di Maro S, Merlino F, Mangoni ML, Luca V, Di Grazia A, Gatti S, Campiglia P, Gomez-Monterrey I, Novellino E, Catania A. 2013. Novel α-MSH Peptide Analogues with Broad Spectrum Antimicrobial

Activity. PLoS One 8:e61614.

60. Gun Lee D, Yub Shin S, Kim D-H, Yeol Seo M, Hyun Kang J, Lee Y, Lyong Kim K, Hahm K-S. 1999. Antifungal mechanism of a cysteine-rich antimicrobial peptide, Ib-AMP1, from Impatiens balsamina against *Candida albicans*.Biotechnology Letters.

61. Brodersen DE, Etzerodt M, Madsen P, Celis JE, Thøgersen HC, Nyborg J, Kjeldgaard M. 1998. EF-hands at atomic resolution: The structure of human psoriasin (S100A7) solved by MAD phasing. Structure 6:477–489.

62. Hein KZ, Takahashi H, Tsumori T, Yasui Y, Nanjoh Y, Toga T, Wu Z, Grötzinger J, Jung S, Wehkamp J, Schroeder BO, Schroeder JM, Morita E. 2015. Disulphide-reduced psoriasin is a human apoptosisinducing broad-spectrum fungicide. Proc Natl Acad Sci U S A 112:13039–13044.

63. Gläser R, Harder J, Lange H, Bartels J, Christophers E, Schröder JM. 2005. Antimicrobial psoriasin (S100A7) protects human skin from *Escherichia coli* infection. Nat Immunol 6:57–64.

64. Kovanda LL, Sullivan SM, Smith LR, Desai A V., Bonate PL, Hope WW. 2019. Population pharmacokinetic modeling of VL-2397, a novel systemic antifungal agent: Analysis of a single- And multiple-ascending-dose study in healthy subjects. Antimicrob Agents Chemother 63.

65. Nakamura I, Ohsumi K, Takeda S, Katsumata K, Matsumoto S, Akamatsu S, Mitori H, Nakai T. 2019. ASp2397 is a novel natural compound that exhibits rapid and potent fungicidal activity against Aspergillus species through a specific transporter. Antimicrob Agents Chemother 63.

66. Machado RJA, Junior LGM, Monteiro NKV, Silva-Júnior AA, Portaro FCV, Barbosa EG, Braga VA, Fernandes-Pedrosa MF. 2015. Homology modeling, vasorelaxant and bradykinin-potentiating activities of a novel hypotensin found in the scorpion venom from *Tityus stigmurus*. Toxicon 101:11–18.

67. Machado RJA, Estrela AB, Nascimento AKL, Melo MMA, Torres-Rêgo M, Lima EO, Rocha HAO, Carvalho E, Silva-Junior AA, Fernandes-Pedrosa MF. 2016. Characterization of TistH, a multifunctional peptide from the scorpion *Tityus stigmurus*: Structure, cytotoxicity and antimicrobial activity. Toxicon 119:362–370.

68. Marques-Neto LM, Trentini MM, das Neves RC, Resende DP, Procopio VO, da Costa AC, Kipnis A, Mortari MR, Schwartz EF, Junqueira-Kipnis AP. 2018. Antimicrobial and chemotactic activity of scorpion-derived peptide, ToAP2, against *Mycobacterium massiliensis*. Toxins (Basel) 10.

69. Guilhelmelli F, Vilela N, Smidt KS, de Oliveira MA, da Cunha Morales Álvares A, Rigonatto MCL, da Silva Costa PH, Tavares AH, Freitas SM de, Nicola AM, Franco OL, Derengowski L da S, Schwartz EF, Mortari MR, Bocca AL, Albuquerque P, Silva-Pereira I. 2016. Activity of Scorpion Venom-Derived Antifungal Peptides against Planktonic Cells of *Candida spp*. and *Cryptococcus neoformans* and *Candida albicans* Biofilms. Front Microbiol 7:1844.

70. Notomista E, Falanga A, Fusco S, Pirone L, Zanfardino A, Galdiero S, Varcamonti M, Pedone E, Contursi P. 2015. The identification of a novel *Sulfolobus islandicus* CAMP-like peptide points to archaeal microorganisms as cell factories for the production of antimicrobial molecules. Microb Cell Fact 14:126.

71. Roscetto E, Contursi P, Vollaro A, Fusco S, Notomista E, Catania MR. 2018. Antifungal and anti-biofilm activity of the first cryptic antimicrobial peptide from an archaeal protein against *Candida spp*. clinical isolates. Sci Rep 8.

72. Park CB, Yi KS, Matsuzaki K, Kim MS, Kim SC. 2000. Structure-activity analysis of buforin II, a histone H2A-derived antimicrobial peptide: The proline hinge is responsible for the cell-penetrating ability of buforin II. Proc Natl Acad Sci U S A 97:8245–8250.

73. Park SC, Kim MH, Hossain MA, Shin SY, Kim Y, Stella L, Wade JD, Park Y, Hahm KS. 2008. Amphipathic α-helical peptide, HP (2-20), and its analogues derived from *Helicobacter pylori*: Pore formation mechanism in various lipid compositions. Biochim Biophys Acta - Biomembr 1778:229–241.

74. Lee DG, Park Y, Kim HN, Kim HK, Kim P II, Choi BH, Hahm KS. 2002. Antifungal mechanism of an antimicrobial peptide, HP (2-20), derived from N-terminus of *Helicobacter pylori* Ribosomal Protein L1 against *Candida albicans*. Biochem Biophys Res Commun 291:1006–1013.

75. Park Y, Hahm KS. 2005. Effects of N- and C-terminal truncation of HP (2-20) from *Helicobacter pylori* ribosomal protein L1 (RPL1) on its anti-microbial activity. Biotechnol Lett 27:193–199.

76. López-Abarrategui C, McBeth C, Mandal SM, Sun ZJ, Heffron G, Alba-Menéndez A, Migliolo L, Reyes-Acosta O, García-Villarino M, Nolasco DO, Falcão R, Cherobim MD, Dias SC, Brandt W, Wessjohann L, Starnbach M, Franco OL, Otero-González AJ. 2015. Cm-p5: An antifungal hydrophilic peptide derived from the coastal mollusk *Cenchritis muricatus*

(Gastropoda: Littorinidae). FASEB J 29:3315–3325.

77. López-Abarrategui C, Alba A, Silva ON, Reyes-Acosta O, Vasconcelos IM, Oliveira JTA, Migliolo L, Costa MP, Costa CR, Silva MRR, Garay HE, Dias SC, Franco OL, Otero-González AJ. 2012. Functional characterization of a synthetic hydrophilic antifungal peptide derived from the marine snail *Cenchritis muricatus*. Biochimie 94:968–974.
